# Acridine Orange: A Review of Novel Applications for Surgical Cancer Imaging and Therapy

**DOI:** 10.3389/fonc.2019.00925

**Published:** 2019-09-24

**Authors:** Vadim A. Byvaltsev, Liudmila A. Bardonova, Naomi R. Onaka, Roman A. Polkin, Sergey V. Ochkal, Valerij V. Shepelev, Marat A. Aliyev, Alexander A. Potapov

**Affiliations:** ^1^Neurosurgery and Innovative Medicine Department, Irkutsk State Medical University, Irkutsk, Russia; ^2^Irkutsk Scientific Center of Surgery and Traumatology, Irkutsk, Russia; ^3^University of Arizona College of Medicine, Phoenix, AZ, United States; ^4^Federal State Autonomous Institution “N. N. Burdenko National Scientific and Practical Center for Neurosurgery” of the Ministry of Healthcare of the Russian Federation, Moscow, Russia

**Keywords:** acridine orange, radiodynamic therapy, photodynamic therapy, intraoperative fluorescence, surgical cancer imaging

## Abstract

**Introduction:** Acridine orange (AO) was first extracted from coal tar in the late nineteenth century and was used as a fluorescent dye. In this paper, we review emergent research about novel applications of AO for fluorescence surgery and cancer therapy.

**Materials and methods:** We performed a systematic search in the MEDLINE, PubMed, Cochrane library, Google Scholar, Embase, Web of Science, and Scopus database using combinations of the term “acridine orange” with the following: “surgical oncology,” “neuropathology,” “microsurgery,” “intraoperative fluorescence,” “confocal microscopy,” “pathology,” “endomicroscopy,” “guidance,” “fluorescence guidance,” “oncology,” “surgery,” “neurooncology,” and “photodynamic therapy.” Peer-reviewed articles published in English were included in this review. We have also scanned references for relevant articles.

**Results:** We have reviewed studies on the various application of AO in microscopy, endomicroscopy, intraoperative fluorescence guidance, photodynamic therapy, sonodynamic therapy, radiodynamic therapy.

**Conclusion:** Although the number of studies on the clinical use of AO is limited, pilot studies have demonstrated the safety and feasibility of its application as an intraoperative fluorescent dye and as a novel photo- and radio-sensitizator. Further clinical studies are necessary to more definitively assess the clinical benefit AO-based fluorescence guidance, therapy for sarcomas, and to establish feasibility of this new approach for the treatment of other tumor types.

## Introduction

Recent advancements in optical cancer imaging have facilitated the development of novel repurposing of known molecular probes for wide-field and microscopic fluorescence-based surgical guidance that have significant potential to positively impact patient care and to transform the field of surgical oncology. Acridine orange (AO) is a common fluorescent dye that has been well known for years. It has recently regained attention as a possible innovative drug for clinical applications in the field of oncology, particularly in cancer imaging and photodynamic therapy.

Acridine orange (AO) is a member of the xanthene class of molecules and shares a common aromatic structure with multiple acridine dyes (1, 2). Acridine dyes were first extracted from coal tar at the end of the nineteenth century. And was then used for a period of time as a dye in the fabric industry as well as for biological application. In 1912, Erlich and Beneda proposed to use acridines as antimalarials, with Browning suggesting use as an antimicrobial agent about a decade on in 1922 (3, 4). During World War I and II, the acridine dyes were widely used as antimicrobials prior to the widespread use of penicillin (4). The application of AO has been studied for bacteria detection in clinical specimens with prokaryotes fluorescing bright orange in low pH buffered media, while other cells produce green background fluorescence (5).

**Figure 1:**
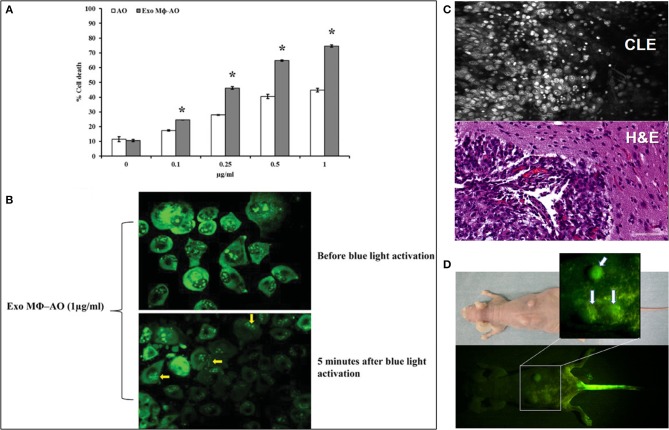
**(A)** Cytotoxic effect of acridine orange (AO)-charged exosomes derived from macrophages (Exo Mϕ-AO) compared to free AO against melanoma cell monolayer by cytofluorimetry assessment. Columns, mean percentages of cell death of two independent experiments run in triplicate; bars indicate standard deviation. *p* < 0.05. Figure adapted with permission from: Iessi et al. (43) (CC BY 4.0). **(B)** Fluorescence microscopy showing the formation of membrane “blebs” in melanoma cells treated with Exo Mϕ-AO (1 lg/ml) after 5 min of exposition to blue light. Figure adapted with permission from: Iessi et al. (43) (CC BY 4.0). **(C)** Upper image—Confocal laser endomicroscopy image of an animal glioblastoma stained with AO. Lower image—corresponding histology image. Image courtesy of Dr. Mark C. Preul. **(D)** Macroscopic fluorescence view of mouse osteosarcoma subcutaneously inoculated in the back of nude mouse after blue light excitation at 2 h after AO injection through the tail vein. Tumors emit green fluorescence (arrows). Injection site of the tail vein also emits green fluorescence Figure adapted with permission from: Kusuzaki et al. (6) (CC-BY 3.0).

The novel research field of acridines is focused on their application in cancer theranostics due to its unique feature of preferential accumulation in the acidic environment of the tumor tissues and strong fluorescent properties (1, 6). AO has been recently explored in several preclinical (Figures 1A,B) and clinical studies for specific tumor cells targeting. As the novel diagnostic and therapeutic applications of AO broaden, we thought to review the existing body of knowledge regarding its potential use in various areas of medicine and outline its place and potential future impact on the rapidly growing area of image-guided cancer management. The goal of this paper is to review the current literature on the use of AO for fluorescence-guided surgery and cancer therapy. This review is focused on the clinical and translational studies that described the use of AO-based therapeutic and relevant diagnostic imaging methods.

## Materials and Methods

### Search Strategy

We performed a literature search in MEDLINE, PubMed, Cochrane library, Google Scholar, Embase, Web of Science, and Scopus databases using the combinations of the term “Acridine” with the following: “surgical oncology,” “neuropathology,” “microsurgery,” “intraoperative fluorescence,” “confocal microscopy,” “pathology,” “endomicroscopy,” “guidance,” “fluorescence guidance,” “oncology,” “surgery,” “neurooncology,” and “photodynamic therapy.” Peer-reviewed articles published in English till September 2018 were included to compile this review. We have also scanned references for relevant articles. Search strategy is presented in Supplement 1.

## Results and Discussion

### Chemical Properties

AO (C17H19N3) is a low molecular weight (265.36 g/mol), weakly basic dye that easily penetrates cell membranes. AO has metachromatic properties and upon excitation with blue light, (~488 nm) emits green fluorescence when in monomer form and orange fluorescence when in dimer form. AO produces orange fluorescence when it binds to RNA and green fluorescence when it binds to DNA. However, this behavior differs significantly in live, apoptotic, and fixed permeabilized cells (6).

In live cells, AO diffuses through the cytoplasmic membrane and is retained in the cellular compartments with low pH, resulting in a bright orange fluorescence of lysosomes when excited with a blue light. AO also intercalates with the cytoplasmic and nuclear RNA molecules and results in a diffuse green fluorescence within all cells (7, 8). AO does not intercalate with DNA of intact living cells (9) and does not accumulate in other non-acidic organelles, such as the mitochondria, endoplasmic reticulum, or Golgi apparatus (7).

In both fixed permeabilized cells and cells undergoing apoptosis, pH compartmentalization is lost. Therefore, AO leaks from acidic lysosomes and diffusely binds to RNA and DNA molecules, resulting in yellow-orange fluorescence in the whole cell (6).

Based on the unique spectral distinctions and differential staining of RNA and DNA molecules, AO can be used to distinguish stages of apoptosis and necroptosis (10). During apoptosis, cells stained with AO demonstrate nuclear shrinkage, cellular fragmentation, and loss of nuclear demarcation, appearing therefore as red cells. Eventually the cellular RNA and red AO emission is lost while fragmented green nucleus remains. In contrast, during necrosis or necroptosis, the orange lysosomal signal is lost due to their disruption and to loss of cellular RNA, while the green signal from smooth and shrunken nuclei remains (11).

Most malignant tumors create an acidic environment due to increased anaerobic glycolysis (Warburg effect). Malignant cells also contain increased amounts of RNA and DNA compared to normal cells. Both factors result in increased accumulation and delayed elimination of AO in tumor cells (12).

### Safety

Acridine dyes have a record of a mutagenic effect on certain types of bacteria, which raises a question about AO safety in humans (13). In 1969, Van Duuren showed that AO is neither an irritant nor a carcinogen to the skin of mice after repeated application. However, three out of twenty mice with skin application of AO developed liver tumors, suggesting systemic absorption of AO (14). In the same study, subcutaneous administration of AO lead to two of thirty mice and one of twenty rats developing tumors at the injection site. (14) In1978, the International Agency for Research on Cancer published the opinion that there was insufficient data or evaluation regarding the carcinogenicity of AO (15).

Systemic administration of AO has been investigated in several animal studies. The LD_50_ for intravenous (IV) administration of AO in mice was determined to be 32 and 36 mg/kg for male and female mice, respectively (16). In determining the dose/toxicity relationship, Tatsuta et al. studied mice with gastric cancer and Tomson et al. studied subcutaneously implanted mammary carcinoma tumors treated with intraperitoneal AO application at a dose of 40 mg/kg and showed effectiveness of photodynamic therapy (PDT) without significant toxic effects (17, 18). Udovich et al. also demonstrated the absence of adverse effects of AO administered intraperitoneally to mice at dose of 0.33 and 3.3 mM for confocal endomicroscopy imaging (19).

In dogs, IV administration of AO at 0.1 mg/kg showed no clinical signs of toxicity and no abnormalities were seen in the blood within 30 days (20). Serum AO levels decreased rapidly within 30 min and were below the detection limit (5 ng/ml) 2 h after intravenous administration (20). In a case report of AO-based PDT in a cat with cutaneous malignant melanoma, no side effects were reported, where AO was administered locally to the surgical site at a dose of 1 μg/ml (21).

Several human studies have demonstrated the initial safety of AO. Kusuzaki et al. reported a pilot study of eight patients with various malignant neoplasms at the terminal stages (two sarcomas, two pancreatic carcinomas, intrahepatic cholangiocarcinoma, lung, renal, and parotid carcinomas) treated with IV administration of AO at 0.5 or 1 mg/kg and low-dose (with 3 or 5 Gy) radiotherapy (RT) (13). This study did not confirm the assumptions about the toxic effects of the AO (13).

Several studies from Japan [*n* = 8 patients from Teneri, Japan (13) and *n* = 51 from Tsu City, Mie, Japan (22)] investigated the local application of AO solution to tumor cavities following tumor resection at the dosage of 1 μg/ml for 5 min. Subsequent analysis of AO-based photodiagnostics (PD), PDT and RT demonstrated no serious immediate or long-term complications (23–26).

In summary, there is still a lack of convincing data regarding the long-term safety and cancerogenic potential of AO. Future studies are needed to demonstrate if potential toxic effects of AO can be balanced by the benefits of AO-based cytoreduction therapies and diagnostics for various tissue organs, routs of administration, and doses.

### Photodiagnostics

#### AO Use for Microscopy

AO has been extensively studied in pathology as a single dye and in combination with other dyes for specimen assessment using fluorescence microscopy. Differential staining of AO has been used to differentiate gliosis and glial neoplasms in human specimens with malignant glial cells demonstrating increased red fluorescence secondary to cytoplasmic RNA accumulation compared to reactive glial cells which remained green (27). AO has been used for mapping of GL261 mouse gliomas on 3-D reconstructed confocal imaging following tissue clearing (28). In this application, glioma regions exhibited increased intensity of fluorescence compared to the dimmer surrounding normal brain (28). Additionally, another study demonstrated the ability of AO to effectively differentiate brain tumor types using confocal microscopy of various brain tumor biopsies rapidly stained with AO (29).

Other applications of acridine orange include the simple and sensitive post-mortem detection of early myocardial infarction—normal myocardium fluoresces a golden brown color and myocardial anoxia/ischemia-damaged cells show a shift toward yellow or yellow-green (30). In the 1980's, Tejada et al. developed a specialized test with sperm AO fluorescence staining to determine male fertility (31). Krishnamurthy et al. showed that staining of breast, lung, kidney, and liver tissues with AO alone and subsequent imaging with confocal laser microscopy practically does not differ in efficiency from staining with H&E (32). Moreover, AO staining was much faster and easier than H&E (32). In yet another application, Wang et al. used AO for rapid non-destructive imaging of whole prostate biopsies using video-rate fluorescence structured illumination microscopy and demonstrated its feasibility as an alternative to destructive pathology (33).

In a study investigating surgical treatment of malignant tumors of the skin, AO was used as a nuclear dye in combination with eosin fluorescence (for labeling cytoplasm), and endogenous reflectance (for labeling collagen and keratin) for tri-modal confocal laser scanning microscopy of the skin samples (34). The authors reported that the novel staining method significantly reduced the time of surgical intervention while providing image quality similar to conventional hematoxylin and eosin staining (34). This finding corroborates with the conclusions drawn by Krishnamurthy regarding the non-inferiority of AO and CLE compared to conventional H&E. Another study reported an automatic pipeline that produces rapid, virtual H&E histology of tissue based on a fast, initial confocal staining of a tissue stained with a combination of AO and sulforhodamine 101, with the extrapolated conclusion that CLE with AO improves time to histological diagnosis over H&E (35). The intraoperative direct analysis that CLE can provide, particularly with AO as a contrast agent, has also been studied in the ophthalmological setting. The authors commented that this method provided a fast *ex vivo* preliminary diagnosis of conjunctival lesions, perhaps representing a novel tool for intraoperative as well as postsurgical management of conjunctival tumors (36).

AO is commonly used as a fluorescent dye to stain live tissues for intraoperative confocal endomicroscopy (Figure 1C), however, *in vivo* topical application of AO has not been investigated for all organs, and particularly in the brain up to this point due to safety concerns, with the less specific fluorescein sodium being used as a fluorescent contrast (37) In mouse brain glioma models and in fresh human biopsies *ex vivo*, AO staining and imaging with the confocal endomicroscope proved successful for differentiation of normal brain and glioma tissue (38, 39). Notably, *in vivo* AO staining was reported for confocal microlaparoscope imaging of ovarian cancer in 45 patients (40). In this study AO was used under FDA Investigational New Drug approval at a dose of ≤ 1 μL of 330 μM/L applied topically to the surface of the ovary just prior to imaging (40). This study concluded that trained surgeons were able to distinguish between normal and malignant ovarian surface epithelium in AO stained optical biopsies and that accuracy was similar during the *ex vivo* and *in vivo* imaging (40). There are two additional publications regarding AO application for the *in vivo* diagnosis of neoplasia in gynecology (41, 42), but currently, no *in vivo* investigation has been published for brain tissue.

#### AO Use in Wide-Field Fluorescence Guidance

After intravenous or topical application and soaking of the resection cavity in AO solution, researchers observed increased accumulation of AO in tumor tissue. This allowed accurate identification of the tumor regions as intense green fluorescence in wide field imaging (using specially designed fluorescence imaging operating microscope). AO fluorescence allowed for better tailoring of the resection area while maintaining the maximum amount of healthy tissue. This technology was used to remove breast tumors (35), soft tissue tumors and sarcomas (26, 44, 45), treatment of skin cancer (34, 46), conjunctival tumors (47), renal tumors, lungs, liver (32).

Considering the rapid development of intraoperative optical imaging tools and significant track of publications regarding AO-based microscopy, AO (similarly to fluorescein sodium and indocyanine green) is a promising topical contrast agent for *in vivo* pathology in humans. However, the long-term safety of topical AO should be established before such a method may be used in surgery. Alternatively, novel, dye-less *in vivo* microscopy methods like optical coherent tomography, RAMAN, and multiphoton reflectance microscopy may make any label-based methods obsolete in the future.

### AO Use in PDT, SDT, and RDT

#### Mechanism of Action

The mechanism of AO-based PDT is based on the production of activated forms of oxygen upon AO fluorescence emission when exposed to blue light excitation (488 nm). As AO binds to the increased amount of RNA in tumor cells and also accumulates in lysosomes, released reactive oxygen species damage lipid membranes, leading to the leakage of lysosomal enzymes, and activation of apoptosis in the tumor cells (12). Myotoxicity was also suggested as another potential mechanism of AO (48).

AO can also increase the production of reactive radicals when exposed to low doses of X-ray radiation (26, 44, 49) or to the ultrasound energy (50). The principles of X-ray radiodynamic therapy (RDT) and sonodynamic therapy (SDT) have gained interest because unlike traditional light-based PDT, it has deeper tissue penetration depth. With RDT however, one energy source simultaneously activates both processes of radiotherapy and PDT, maximizing the synergetic treatment effect (49, 51). RDT with AO shows a similar effect on tumor cells when compared to PDT (52).

#### Preclinical Studies

One of the earliest studies of PDT with AO by Tomson et al. in the mouse model of undifferentiated carcinoma showed tumor resolution after PDT with AO in 90% of mice (18). Another early study by Ishikawa et al. which suggested the usefulness of PDT investigated photodynamic inactivation of argon laser with topical use of AO in the treatment of bladder cancer. Human bladder cancer cells (MGH-U1) were stained with AO and irradiated with argon laser with wave length of 496.5 nm and the intensity at the sample position about 4.0 mW/cm^2^. The result shows that argon laser at the low intensity and with short irradiation time has a sufficient cytocidal effect (53). Satonaka et al. studied the AO-based PDT of pulmonary metastases of osteosarcomas in mice and demonstrated its feasibility (54) (Figure 1D). Interestingly, AO alone and AO-PDT inhibited invasion and metastases growth (54).

AO-based PDT and RDT has been advanced to clinical studies for the treatment of sarcomas (osteosarcomas and rhabdomyosarcomas) (1, 7, 12, 23, 25, 55), while its efficacy for brain tumors has been assessed only in preclinical studies. Osman et al. investigated the efficacy of AO-based PDT of glioma cells *in vitro* (56). Treatment of cultured glioblastoma cells with AO at a concentration of 0.001 mg/mL followed by 10 or 30 min incubation with white unfiltered light from light-emitting diode bulbs demonstrated dramatic cytostatic and cytocidal effects and almost complete eradication of glioblastoma cells in 72 h (56).

#### Local AO Administration

Matsubara et al. investigated the combined use of AO-based PD, followed by PDT and RDT for the treatment of rhabdomyosarcoma (57). After subtotal resection of the tumor, the area of operation was filled with a solution of AO at a concentration of 1 μg/ml and after 5 min, the excess dye was washed off with saline. The first step included the use of wide-field fluorescence microscopy of the surgical area. Authors used a customized operating microscope (Carl Zeiss Co., Ltd, Oberkochen, Germany) with a xenon lamp equipped with an excitation filter (450–490 nm) resulting in a blue excitation light and a long-pass filter (>520 nm) for detection of the green fluorescence emitted by the tumor. After staining, a more thorough curettage of the area was performed until the green glow disappeared. The next step included AO-PDT, a 10 min exposure of the resection cavity to unfiltered light from a xenon lamp with a brightness of 100,000 lx mounted on an operating microscope. After that, a layer of sutures was applied to the wound without rinsing the AO solution. The patient was then transferred to the radiotherapy room where an X-ray exposure was performed (5 Gy) (57).

Matsubara et al. reported long-term results of AO-based PD followed by PDT and RDT in 18 patients from 1996 to 2008, showing that the method allows preservation of more healthy tissue during resection (26, 44). This advantage helped to preserve functionality of the limbs due to more tailored resection compared to the standard wide margin resection method without significant differences in the recurrence rate.

#### Systemic AO Administration

There was only one study found on the AO-based RDT with systemic administration of AO which included treatment of 8 patients with end-stage cancers. In this study, two patients had sarcomas, two had pancreatic cancer, and one patient each had intrahepatic cholangiocarcinoma, renal, lung, and parotid cancer. (13). Two hours after 1 mg/kg AO was administered IV, the patients received 3–5 Gy X-ray irradiation 3 times within a 3 week period. In 3 out of 5 patients who completed the full course of the proposed therapy, tumors reduced in size and symptoms improved. Notably, this study demonstrated the absence of a toxic effect on the body with the introduction of therapeutic doses of the drug (13).

While some patients benefited in these small pilot studies, further clinical investigation is necessary to assess if the observed cytocidal effect could be achieved in other types of cancer. However, the absence of serious side effects of AO, coupled with the likely cytolytic effect of RDT, together with the radiation, opens up new horizons for exploring its potential in other cancers.

## Conclusions and Future Directions

The use of AO in oncology represents a novel approach but the number of studies is currently limited, likely due to the historical concerns about AO safety. More studies are necessary to identify tumor types and stages where application of AO can be useful to assess its clinical value and identify potential side effects.

There are several promising areas where AO may be beneficial. First, it is worth noting the possibility of macro and confocal fluorescence microscopy for *in vivo* fluorescence guidance during surgical resection. The effect of AO fluorescence guidance has not yet been studied in brain tumors and should be tested in comparison with other fluorescence guidance techniques such as 5-aminolevulinic acid, fluorescein sodium, and indocyanine green, which are already available and have a safety record and established efficacy.

Of particular interest is the effect of intravenously administered AO on tumor cells when exposed to small doses of radiation. If successful, this could dramatically change the approach for cancer treatment. AO-based RDT treatment of distant metastasis is another promising area of application (54). AO fluorescence guidance combined with PDT, SDT, or RDT can be potentially applied to all organs for the surgical treatment of tumor lesions, including brain tumor surgery. Brain gliomas are notorious for their infiltrative growth and thus the possibility of selective killing of any residual tumor cells after surgical resection is encouraging and compelling. However, there are also considerable limitations in that the blood-tumor-brain-barrier at the periphery of the gliomas might decrease efficacy of AO delivery, decreasing its photosensitizing effect (58). It should also be noted that AO is one among many drugs (including 5-ALA) that are in development for PDT, SDA, or RDT for the treatment of various cancers and their comparative efficacy for various cancers is yet to be established (59–63). Future drug developments include nanoparticles and targeted multipurpose theranostic drugs which can be used for intraoperative guidance and subsequent precision treatment of any microscopic residual tumor (36).

AO has demonstrated utility in multiple settings up to this point in time with the majority of applications being *ex vivo*. Although use of AO is not new with practical applications from urology, gynecology, to ophthalmology detailed, repurposing AO for possible specific tumor labeling in the field of neurosurgery has been minimally explored. While there is historical evidence of potentially detrimental health effects of AO dating back to the late 1960s, there is a small body of growing literature in the time since that points to its potential uses *in vivo* without toxic systemic effects. With the evidence presented in this review regarding the role of AO in tumor identification, we are especially interested with respect to brain lesions what AO can possibly provide in terms of specific fluorescent tumor labeling. More data is needed to determine the safety of AO *in vivo* and its future applications, particularly in neurosurgery.

## Author Contributions

VB, LB, NO, RP, SO, VS, MA, and AP researched the data, wrote the manuscript, generated the figures, provided participation in the discussion of the content, and revised the review.

### Conflict of Interest Statement

The authors declare that the research was conducted in the absence of any commercial or financial relationships that could be construed as a potential conflict of interest.
