# Supplementary material for: Acridine Orange: A Review of Novel Applications for Surgical Cancer Imaging and Therapy
Source: Front Oncol. 2019 Sep 24;9:925. doi: 10.3389/fonc.2019.00925 (PMC6769070; doi:10.3389/fonc.2019.00925)
Supplement: Supplementary file 1 [file Table_1.docx]

SUPPLEMENT 1

Flowchart depicting study selection process.

Title/Abstract exclusion
(n =3295)

Studies included in quantitative synthesis
(n =53)

Full-text articles assessed for eligibility
(n =105)

Records identified through database searching
(n =3400)

Full-text articles excluded:
(n =52)
